# Advances in 3D Bioprinting for Corneal Regeneration

**DOI:** 10.3390/gels11060422

**Published:** 2025-05-31

**Authors:** Juan Hernández, Nicolás Santos, Manuel Ahumada

**Affiliations:** 1 Escuela de Tecnología Médica, Facultad de Ciencias de la Vida, Universidad Viña del Mar, Viña del Mar 2572007, Chile; 2 Institut Químic de Sarrià, Universitat Ramon Llull, 08017 Barcelona, Spain; nicolassantosq@iqs.url.edu; 3 Centro de Nanotecnología Aplicada, Facultad de Ciencias, Ingeniería y Tecnología, Universidad Mayor, Santiago 8580745, Chile; 4 Escuela de Biotecnología, Facultad de Ciencias, Ingeniería y Tecnología, Universidad Mayor, Santiago 8580745, Chile

**Keywords:** bioprinting, biocompatible materials, cornea, corneal transplantation, tissue engineering

## Abstract

Worldwide, millions of people suffer from visual impairments, ranging from partial to total blindness, with far-reaching consequences on personal, societal, and governmental levels. Corneal-related issues are among the leading causes of blindness, with corneal transplantation (keratoplasty) being the primary treatment. However, the demand for donor tissues far exceeds supply. The rise of printing technologies marks a revolution in tissue engineering, with 3D bioprinting at the forefront of developing innovative tissue repair and replacement solutions. The cornea emerges as an ideal candidate for this technology due to its distinct layers (epithelium, stroma, and endothelium). From a materials engineering standpoint, these layers resemble a hydrogel structure that facilitates fabrication. This review explores advancements in 3D bioprinting, focusing on the methodologies developed for corneal tissue engineering. It highlights design and construction aspects, including biomechanical and biocompatibility properties essential for creating synthetic implants and corneal scaffolds through bioprinting. Additionally, the review discusses the challenges and opportunities that could further drive innovation in tissue engineering.

## 1. Introduction

In 2019, 2.2 billion people worldwide were experiencing some degree of visual impairment; around half of them were suffering from conditions that could have been prevented or were waiting for treatment, according to the World Health Organization. Among those, 950 million people had some impairment related to the cornea, the causes of which (infectious, nutritional, inflammatory, heritable, degenerative, traumatic, or iatrogenic) are known to be preventable and treatable [1]. Yet, under corneal blindness conditions, the only possible treatment is transplantation. However, despite existing clinical interventions, there is limited access to corneal graft tissue (unavailable for over 95% of patients) [2], with a further risk of posterior rejection of the transplantation caused by the immune response, which can affect “the survival of the cornea transplanted” [3]. Therefore, the use of immunosuppressive agents is required to increase the five-year success rate of corneal implants to up to 90% [4]. Such conditions have given rise to urgency to massify/develop alternative solutions to solve corneal blindness, considering corneal properties and patient health.

The cornea is a transparent, avascular, and sensitive tissue found in the anterior sixth of the eyeball [5,6]. It fulfills two essential functions: (a) it serves as a mechanical protective barrier and (b) it exhibits transparency and light refraction characteristics [7]. Upon irreversible transparency loss, keratoplasty is the most widely recommended treatment to restore vision [8,9], replacing unhealthy tissue with a healthy one from a cornea donor. Although this procedure has demonstrated promising results and is particularly associated with corneal “immune privilege” [10], there is a shortage in the number of available corneas around the world, i.e., a ratio of 1:70 [2]. Keratoplasties (in all their forms), like other transplants, can present complications or graft rejection [11], requiring new interventions and/or underlining the need for biomaterials such as keratoprostheses or artificial corneas to restore vision [12,13].

Three types of keratoprosthesis can be found: (a) those composed of solid (metal) and synthetic materials, such as polymethylmethacrylate (PMMA) lenses, like the Boston KPro; (b) those using the patient’s bone or dental tissue, i.e., osteodontokeratoprosthesis (OOKP) and osteokeratoprosthesis (OKP), along with a PMMA lens; and (c) AlphaCor, created from a flexible material composed of poly-2-hydroxyethyl methacrylate (PHEMA) [14]. Despite being an excellent alternative to keratoplasty, keratoprostheses present some complications. For instance, infections, extrusion of the implant and total loss of vision have been reported in Boston KPro users [15]; the development of glaucoma, ulcers, and mucosal overgrowth on the OOKP lens has been observed [16]; and, in the case of AlphaCor, melting of the corneal stroma, membrane formation, glaucoma, and implant extrusion have been reported [17]. Through tissue bioengineering, artificial corneas are being developed for histologic regeneration or replacement [18]. With this goal, synthetic polymers such as poly(ethylene glycol) (PEG) [19,20], polycaprolactone (PCL) [21], poly(vinyl alcohol) (PVA) [22,23], and poly(lactic-co-glycolic acid (PLGA) [24], as well as natural macromolecules such as collagen [25], gelatin [26], alginate [27], and hyaluronic acid [28,29], to name a few, have been employed to develop such corneal replacements. Additionally, decellularized corneas [30] and self-assembled molecules [31,32] have been used. One example of the advancements in this area is the study by Fagerholm et al., who used recombinant human collagen (RHC) to develop synthetic lamellar corneas, subsequently undertaking a phase 1 clinical trial, evaluated at 2 and 4 years [33,34]. In the second year, the implants were stable and avascular, with improvement in visual acuity being reported in six patients. After 4 years, significant corneal regeneration was observed, resulting in an improved vision by five Snellen visual acuity lines. Another clinical trial conducted by Islam et al. [35] used RHC implants with 2-methacryloyloxyethyl phosphorylcholine as a supporting polymer in six high-risk transplant rejection patients. The results showed that the patients recovered corneal sensitivity, although only 50% reported visual improvements.

Recently, the tissue engineering field has adopted 3D printing technology to build three-dimensional organ and tissue structures, called 3D bioprinting. This process has been defined as “a computer-assisted transfer process for the simultaneous ‘writing’ of living cells and biomaterials with a layer-by-layer stacking prescribed for the manufacturing of bioengineered constructs in tissue engineering, regenerative medicine, or other studies.” [36]. This technology has created artificial organs such as miniature hearts, kidney tissues, ovaries, livers, ears, pancreas, skin, noses, and corneas, all designed by computer models [37,38]. In the case of corneal printing, bioprinting is a valuable tool for creating this tissue due to its precision in the construction of shapes, surface roughness, and mechanical resistance, as well as the on-demand laminar and cellular distribution in the development of in vitro models [39]. This review introduces 3D bioprinting, offering an overview of several bioprinted corneal tissues that have been developed in recent studies. It also highlights the main types of related technologies currently available, including materials, cells, and tests used for their characterization. Furthermore, biomechanical, optical, and bio-requirements are explored to find the optimal form of corneal replacement.

## 2. Three-Dimensional Bioprinting

### 2.1. Overview

Three-dimensional printing technology originated in the 80s and has since diversified into various areas; however, its most notable technological advancements and applications began in the current century. In economic terms, it represents a growing market of USD 9.9 billion in 2018, with forecasts indicating that it could reach USD 34.8 billion by 2024 [40]. As mentioned, various types of 3D printers are differentiated by their techniques, such as MultiJet fusion, stereolithography, selective laser sintering, deposition modeling casting, direct metal laser sintering, and digital light processing [41,42]. Its application in the medical field has emerged over the last decade with the development of 3D bioprinting [40,41].

### 2.2. Bioprinting Methods

**Figure 1:**
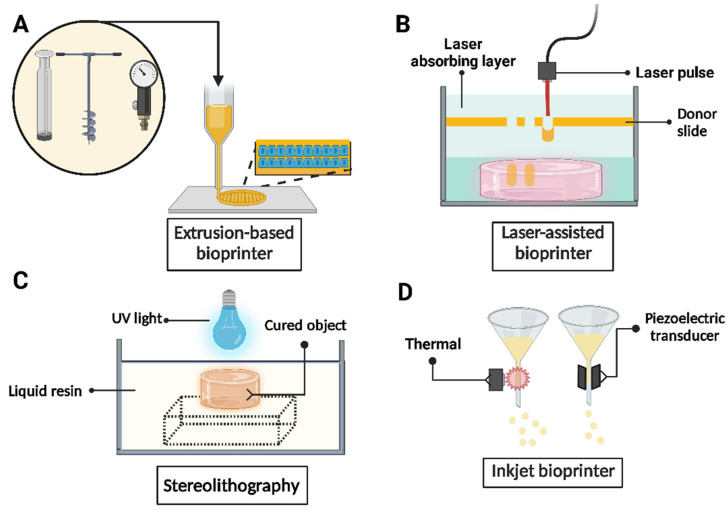
Schematic representation of the principal 3D bioprinting methodologies. (**A**) Extrusion-based bioprinting; (**B**) laser-assisted bioprinting; (**C**) stereolithography; and (**D**) inkjet/droplet bioprinting.

Bioprinting is the material transfer process that models and assembles molecules, cells, tissues, and biodegradable biomaterials, transforming them into biologically relevant materials with a prescribed organization to achieve one or more biological functions [43]. Moreover, it has proven helpful in drug response evaluation and tissue morphogenesis [44]. Bioprinting utilizes computer systems (both software and hardware) to incorporate cells and other bioactive elements, which are spatially distributed layer by layer or point by point in a controlled manner. This approach recreates the structure, architecture, and function of organs and tissues, offering significant advantages, such as the ability to digitally define the tissue construct of interest and accurately reproduce the physical characteristics of complex biological structures with precise geometric control [45,46]. The elements used to create these constructs are collectively called bioinks [47]. The support matrix for these bioinks can be made from natural polymers or derived from gelatin, collagen, silk [48], hydrazone with hyaluronic acid [49], and alginate, or synthetic ones such as lactic acid polymers (PLAs) or polycaprolactone (PCL), to name a few [44,50]. Within a controlled micro-environment, these bioinks can mimic the native extracellular matrix, providing signaling hints and binding sites to facilitate targeted cell migration, proliferation, and differentiation to promote tissue renovation [51]. Thus, a bioink is a biofunctional material that contains cells and provides an extracellular matrix to withstand the mechanical and thermal stress of the printing process. This process can be achieved through various methods, including injection, extrusion, laser assistance, and stereolithography (see Figure 1) [41,52]. Furthermore, when sensitive materials are considered, it is possible to adjust the printing parameters or external conditions over time to control the maturation of the printed tissue, a technique known as 4D bioprinting [53,54]. Additionally, when the host is the bioreactor, it is referred to as in situ bioprinting [42].

### 2.3. Tissue Bioprinting

Generally, the creation of tissue by bioprinting can be approached through two different methods [44]. The first relies on 3D molds filled with an acellular biomaterial onto which cells are loaded. Reiffel et al. (2013) provided an example of this method by designing a human ear mold filled with collagen dissolution and seeded bovine chondrocytes to reconstruct the ear of a pediatric patient with microtia [55]. The second approach involves incorporating cells and/or cell aggregates with the biomaterial for the final design. For instance, Norotte et al. (2009) utilized this method to construct vascular tissues [56]. Despite these two approaches, the creation of 3D biological constructs has been described as a three-step process [41]:

(i) Pre-processing: This involves planning and designing the 3D structure by generating images and computer-aided design (CAD) of the object (organ or tissue) to be processed.
(ii) Bioink preparation and printing: This comprises the selection of biomaterials for their preparation and choice of printing methodology.
(iii) Post-processing: This involves the maturation of the cell-laden construct to enhance tissue development, including biomimetics or biomimicry, autonomous self-assembly, and tissue building blocks.

For the first step, diagnostic imaging tools such as computed tomography (CT), magnetic resonance imaging (MRI), X-rays, or ultrasound are utilized to create three-dimensional CAD models. Subsequently, the 3D model is segmented (sliced) into layers to generate a coordinate-based design (G-code). This coordinate system comprises the instructions that the bioprinter uses to move the printhead or nozzle along the three Cartesian axes, the movement speed, temperatures, and the bioink volumes at each construction point. In the second step, the selection of the biomaterial(s), crosslinker, and growth factors, to name a few, is carried out to replicate the native tissue’s properties [57]. Finally, the third step corresponds to the conditions under which the biomaterial is preserved to promote the maturation and growth of the printed construct, mediated by a bioreactor, and to monitor the conditions, maintaining the physicochemical properties and biological environment applied over the target zone [58].

### 2.4. Corneal Bioprinting

**Figure 2:**
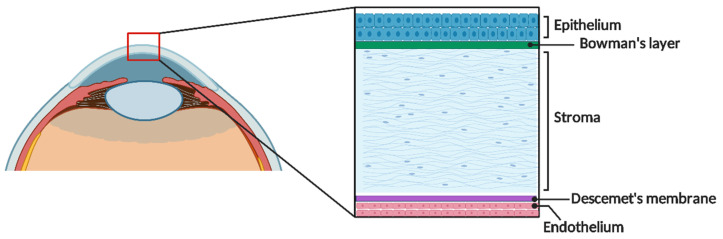
Schematic representation of the cornea and the layers that compose it.

The cornea (Figure 2) possesses anatomical and physiological features that provide significant advantages for its development in 3D bioprinting. Among these advantages are its lack of blood vessels, which reduces the likelihood of immune rejection; its simple cellular composition (epithelial, stromal, and endothelial), making it relatively easy to print compared to more complex tissues; its uniform curvature, which simplifies design and printing as the final shape is more predictable; and its primary composition of collagen, which facilitates the use of a wide range of biopolymers for printing, such as collagen, gelatin, and fibrin [59,60,61,62].

The first reference to using 3D bioprinting for cornea replacement appeared as an abstract in a supplement to the Cornea Journal, written by Hong Kyun Kim in 2014. In this work, the author established a prototype 3D printer to create a stromal substitute from a mixture of fibrinogens and a hyaluronic acid gel, on which limbal keratocytes were seeded. The results indicated that the imprinted substitutes were morphologically and biologically comparable to in vivo human stromal tissue [63]. This research initiated the development of various investigations focused on generating 3D-printed corneal implants.

### 2.5. Bioinks Designing for Corneal Applications

**Figure 3:**
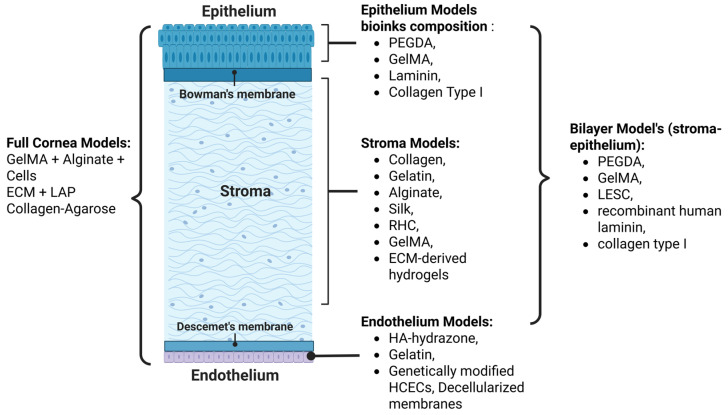
Schematic representation of bioink components commonly used for the bioprinting of corneal tissues. The distribution of specific materials is organized according to the main anatomical layers of the cornea: epithelium, stroma, and endothelium.

Several 3D bioprinting results for corneal tissue repair or replacement have been described in the literature, highlighting the relevance of biological tissue and experimental conditions. However, as previously mentioned, the key to bioprinting lies in the choice of bioink. In this regard, various biomacromolecules serving as components for the bioink matrix have been discussed, with Figure 3 summarizing the main bioink’s components used for different corneal layers, providing an overview that bridges the transition from general bioprinting concepts to specific applications in corneal tissue engineering.

**Figure 4:**
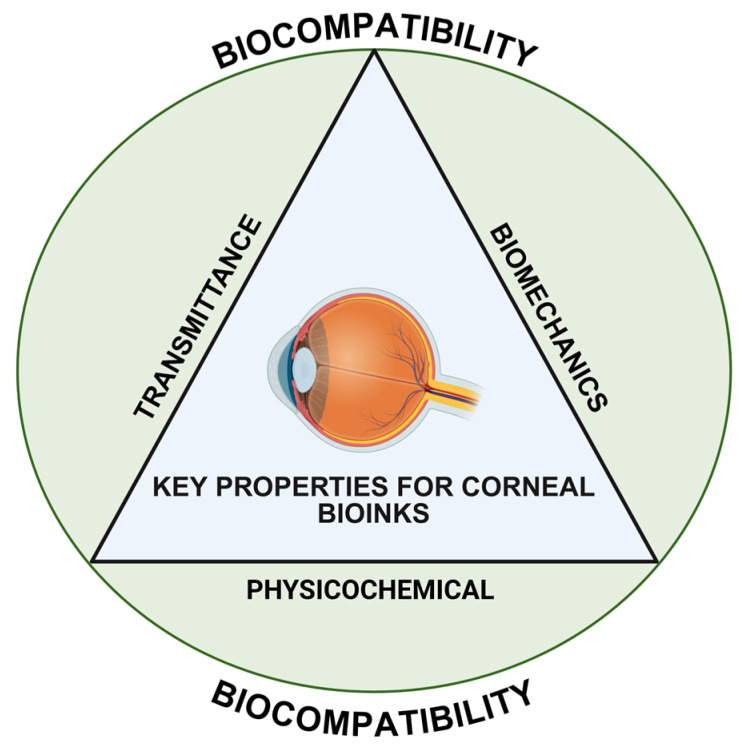
Illustration of the essential properties required to develop biocompatible corneal 3D-bioprinted implants.

Similarly, three critical aspects must be considered when developing a bioink for corneal tissue: the material’s biomechanical, physicochemical, and optical properties (Figure 4). Consequently, the overall biocompatibility of the construct is determined by integrating these parameters, which ultimately favor cell viability and implant functionality, while ensuring structural integrity, chemical stability, and appropriate light transmission [64,65]. For instance, a physicochemical property such as swelling behavior, which refers to the water content of the cornea/biomaterial, strongly influences the implant’s transparency and mechanical performance, as well as ensuring intraocular pressure equilibrium and the dimensional stability of the 3D-bioprinted structure [66].

It is important to note that the biocompatibility of biomaterials extends beyond merely determining the absence of cytotoxicity or a favorable immune response. Further efforts must be made to ensure proper biofunctionality of the 3D-bioprinted materials. Key considerations include the material’s ability to interact with host tissue, promote cell migration, and preserve the physiological microenvironment, essential for regenerating native corneal tissue [67,68,69]. The following sections will further explore these properties, emphasizing their significance in the bioink design process for corneal bioprinting applications.

### 2.6. Biomechanical Requirements

Despite efforts to generate new materials that mimic a native cornea, corneal grafts must possess mechanical properties such as hardness, stiffness, and elasticity to support the wounded tissue while allowing cell integration with the host tissue, thereby enhancing biocompatibility. Consequently, scaffold design typically combines synthetic and natural polymers [70]. Nevertheless, replicating the biomechanical properties of corneal tissue remains a challenge.

Considering this biomechanical challenge, alongside the standard strain–stress curve measurements, evaluating the various degrees of elastic deformations produced by corneal tissue at low-stress values is essential, as failures can lead to corneal stiffness [71]. Regarding the Young’s modulus (“E”, stress/strain curve), measuring stiffness and its variation with pressure can enhance the understanding of corneal tonometry. In this regard, orientation on measurements is a relevant parameter. For instance, it can be observed that measurements at the low-strain tangent modulus with a strain of less than 20% (NT: 1.17 ± 0.43 MPa; SI: 1.32 ± 0.50 MPa) can be found. Meanwhile, for a strain between 35% and 55% (NT: 43.59 ± 7.96 MPa; SI: 51.26 ± 8.23 MPa), NT refers to the nasal–temporal and SI in the superior–inferior anatomical directions [72]. Nevertheless, these parameters can vary across different studies, suggesting that they can serve as a reference for future researchers [73].

Further analysis related to the biomechanical characterization of corneal tissue revealed linear behavior regarding the Young’s modulus and applied posterior pressure, which was evaluated from 0 to 75 mmHg of pressure (E–p relationship). The results indicated significant variation concerning the age of the human corneas tested. However, all groups exhibited an E over 600 kPa at a pressure of 75 mmHg, facilitating the understanding of corneal stiffness at lower pressures [74].

Research related to bioprinting enables the simulation of corneal curvature, as noted by Gingras and colleagues (2024). The authors discovered that a bioink composed of natural polymers (alginate and gelatin) has a compressive modulus of 23.7 ± 1.7 kPa (secant) and 42.2 ± 9.0 kPa (tangent) (at 20% strain). In comparison, the native corneal compressive stiffness exceeds 300 kPa, which underscores the challenge of employing natural polymers, as previously mentioned [75].

To address this gap, researchers have introduced corneal extracellular matrix (ECM) components into bioinks for 3D bioprinting [62]. Due to their properties, such as stiffness, they play a significant role in the biomechanical properties of corneal tissue and, consequently, influence the mechanical phenotype, which consists of highly ordered collagen (Col I and Col V) fibers, glycosaminoglycans and proteoglycans (with lumican and keratocan predominating), elastic fibrils, basement membranes, and matrix proteins [76,77]. For instance, Puistola and colleagues’ (2024) research incorporated ECM into a bioink based on hyaluronic acid modified with an aldehyde group for posterior crosslinking. The bioink was applied using extrusion-based 3D printing, and the resulting material retained the key corneal stroma-specific proteins, allowing it to mimic the native corneal microstructure. In addition, the bioink promoted structural integrity and transparency, producing a cellular morphology similar to that of the native corneal stroma. Nevertheless, further mechanical characterization is still required [78]. Other studies related to ECM-derived bioinks were conducted by Kim and colleagues (2019), who demonstrated that the 3D printing technique provides a cornea-mimicking microenvironment. Various collagen fibril concentrations were tested (from 0.5 to 2 wt%), showing a logarithmic modulus conversion behavior around 100 kPa. Moreover, the sample with higher fibril content exhibited a gelation time 8.7 times shorter than the 0.5 wt% due to the readily crosslinked bioink, a result consistent with the increased viscosity of the material [79].

### 2.7. Optical Requirements

In a healthy human cornea, visible light transmittance is between 70 and 90% in the 400–700 nm range [80]. In addition, the refractive index ranges from 1.335 to 1.4391, ensuring minimal distortion of incoming light [81]. Consequently, when engineering corneal scaffolds, these optical characteristics correspond to a critical feature for corneal regeneration and the potential applicability of these biomaterials [82].

Kilic Bektas and Hasirci (2018) developed photopolymerizable GelMA hydrogels. This material initially exhibited over 90% transmittance across the visible spectrum, closely matching the native cornea. Furthermore, adding human keratocytes did not significantly affect the transparency, as the cells remained well-distributed and actively synthesized the stromal extracellular matrix. Thus, this approach demonstrated high initial optical clarity [83]. Meanwhile, GelMA-HA-based bioinks for 3D-printed corneal scaffolds have shown different transmittance behavior. For instance, Wang and colleagues (2022) reported that their acellular construct presented a 40.7% transmittance at 400 nm and 63.2% at 700 nm, values significantly lower than those found in the native cornea (74.8% and 91.2%) and the GelMA hydrogels previously published by Kilic Bektas and Hasirci (2018). However, after 90 days of corneal stromal (CS) cell culture, transparency improved (to 69.8% at 400 nm and 74.0% at 700 nm) due to cell-driven remodeling and extracellular matrix deposition, although it still fell short of native corneal transparency. These findings suggest that, while 3D bioprinting enables greater structural control, prolonged cultivation times may be necessary to fully match the optical properties of natural corneal tissue [83]. Furthermore, GelMA-HA allowed ECM deposition and remodeling, enhancing the biomaterial’s bioactivity, while GelMA alone maintained transparency but did not significantly change due to cell activity. Additionally, 3D printing allows precise control over complex structures, facilitating cell infiltration within the scaffold [84]. On the other hand, Chand et al. (2025) provide insights into optimizing the structural integrity and transparency of GelMA-based bioprinted corneal implants. To achieve this, oxidized carboxymethylcellulose (OxiCMC) was added to enhance mechanical properties without compromising transparency. Additionally, tartrazine improved printing resolution, and after four washes, the scaffolds achieved transmittance values comparable to the native cornea (75% at 400 nm, 91% at 700 nm) [85].

Under this general protocol, various biomaterials for 3D bioprinting have been developed as a potential solution for repairing multiple tissues. Similarly, such advances have also been applied to the visual organ, with the cornea being the most studied, while the posterior pole (retina) has remained theoretical [58]. Current reports on corneal bioprinting include the epithelium, stroma, endothelium, and combinations of these components. Some of the most notable achievements in 3D bioprinting related to corneal tissue and its layers are now presented.

## 3. Advancements in Corneal 3D Bioprinting

### 3.1. The Epithelium in 3D Bioprinting

**Figure 5:**
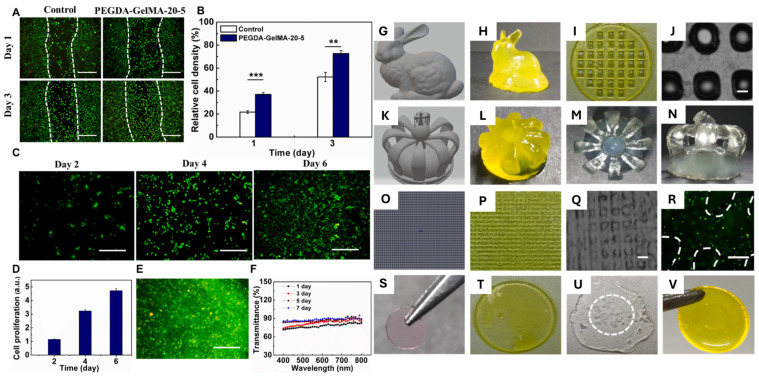
Cytocompatibility and printability properties of PEGDA-GelMA hydrogels for epithelial applications. (**A**–**F**) In vitro cytocompatibility analysis of rabbit corneal epithelial cells (rCECs) seeded on/or encapsulated within PEGDA-GelMA-20-5 hydrogels, demonstrating high viability and proliferation; **, *** represent *p* < 0.05, and *p* < 0.01, respectively. (**G**–**V**) Macroscopic appearance of a 3D printability evaluation of hydrogel material for corneal scaffold. Reproduced from [67] with permission of Elsevier B.V. under CC BY-NC-ND 4.0 license.

He et al. (2022) developed an epithelial/stromal bilayer biomimetic scaffold composed of long-chain polyethylene glycol diacrylate (PEGDA) at various concentrations (10, 15, and 20 wt%) and gelatin methacrylate (GelMA) at 5 wt%. Utilizing a stereolithography printing methodology, the incorporation of PEGDA in increasing concentrations improved the mechanical resistance of the developed hydrogel through the photo-crosslinking of the polymer with GelMA. In vitro cytotoxicity assays of the biomaterial were performed on L929 mouse fibroblasts and rabbit corneal epithelial cells (rCECs), demonstrating favorable cell proliferation and integration with the receptor corneal bed, promoting stromal regeneration without inducing corneal scarring [67]. Figure 5 illustrates the previously described cytocompatibility results and the printability properties of PEGDA-GelMA hydrogels for epithelial applications.

**Figure 6:**
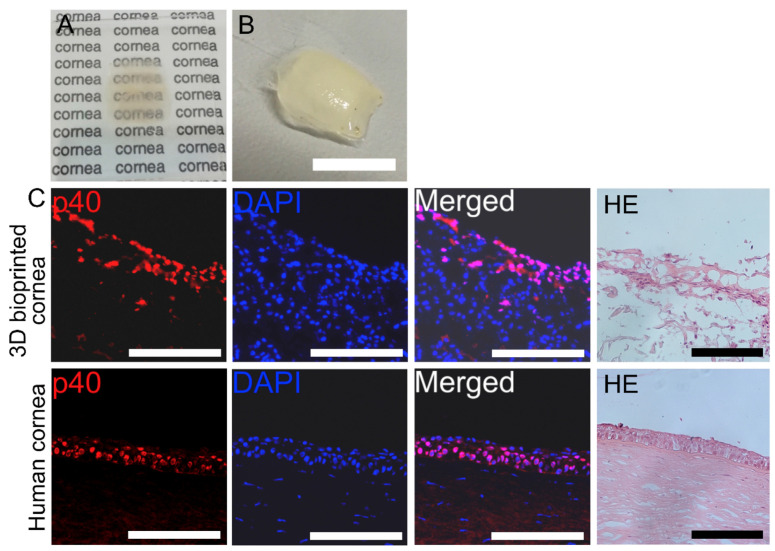
Multilayered 3D-bioprinted corneal epithelial constructs using hESC-LESCs. (**A**,**B**) Bioink transparency and raw form, scale bar = 10 mm. (**C**) Fluorescent staining and histological analysis revealing structural similarities between the printed construct and native human cornea, scale bar = 200 μm. Reproduced from [62] with permission of Elsevier B.V under CC BY-NC-ND 4.0 license.

Sorkio et al. (2018) used a laser-assisted bioprinter to create three structures that mimicked the human cornea: epithelium, stroma, and a combination of both. Primarily related to the epithelium, limbal epithelial stem cells (LESCs) derived from human embryonic cells were utilized. The bioink contained recombinant human laminin, a major component of the limbal epithelial cells’ basal membrane, and human collagen type I, a central component of the corneal stroma (see Figure 6). The results demonstrated cell viability and proliferation of the stem cells as well as the ability of the imprinted LESCs to form a stratified epithelium resembling a mature cornea and expressing specific proteins [62].

Integrating polymers and macromolecules in developing biomimetic scaffolds emphasizes the importance of designing materials with physicochemical properties similar to those of native tissue. These materials should promote cell adhesion and proliferation that mimic the native environment, working synergistically to create a scaffold capable of facilitating epithelial regeneration within the cornea.

### 3.2. The Stroma in 3D Bioprinting

**Figure 7:**
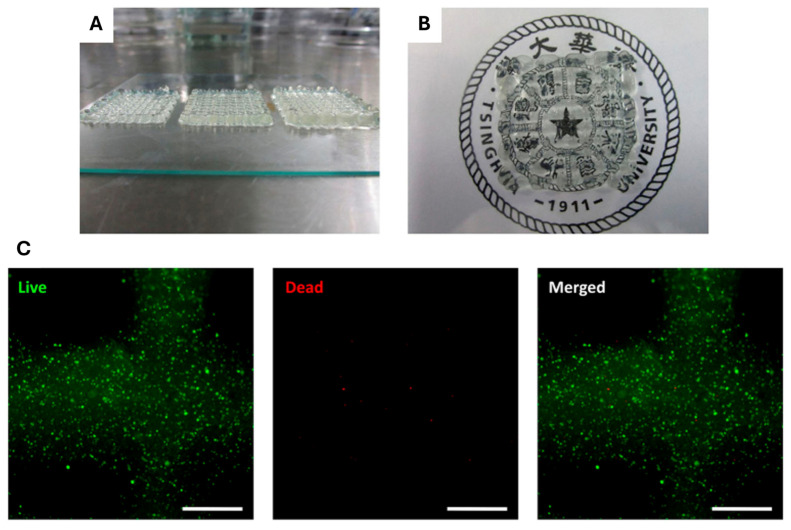
Optical transparency in bioprinted stromal constructs. Visual assessment of scaffolds with varying numbers of printed layers in (**A**), transparency in (**B**), and cell viability (live/dead staining) after printing in (**C**), scale bar = 500 μm. Reproduced from [44] with permission of Springer Nature under CC BY 4.0 license.

As previously mentioned, Sorkio et al. (2018) used a laser-assisted bioprinter to create a human cornea mimic (reassembling the epithelium and stroma). Regarding the stroma, adipose-derived stem cells (ADSCs) were combined with recombinant human laminin and human collagen type I as bioinks. An increase in cell proliferation and viability was observed, dependent on the material’s thickness (cell migration). The presence of collagen type I and human plasma in the stromal matrix was verified, along with the Ki67 marker (used to identify cells in the active proliferation phase of the cell cycle). The cellular organization was arranged horizontally, resembling that of a native cornea, and aligned orthogonally between adjacent layers. At 14 days, the graft thickness decreased to around 300 µm, indicating degradation of the printed matrix [62]. Wu et al. (2016) presented promising results when making a laminate printed from a bioink composed of collagen hydrogel, gelatin, and alginate combined with human corneal epithelium cells (see Figure 7) [44]. The extrusion method developed a construct of multiple sheets in the form of grid-like layers that were 0.1 mm thick. The layers were crosslinked with 3% calcium chloride to stabilize the material. Subsequently, the biomaterial was cultured using DMEM/F12 medium for up to two weeks. The final construct was transparent to visible light, transmitting over 60% for wavelengths up to 630 nm. Morphological stability was observed when stacking 10 to 20 sheets, alongside cellular viability exceeding 90%. Additionally, adjusting the molar ratio between sodium alginate and sodium citrate in the culture medium allowed it to control the biomaterial degradation without affecting cell proliferation, thus regulating the desired response regarding mechanical properties and cell interaction.

**Figure 8:**
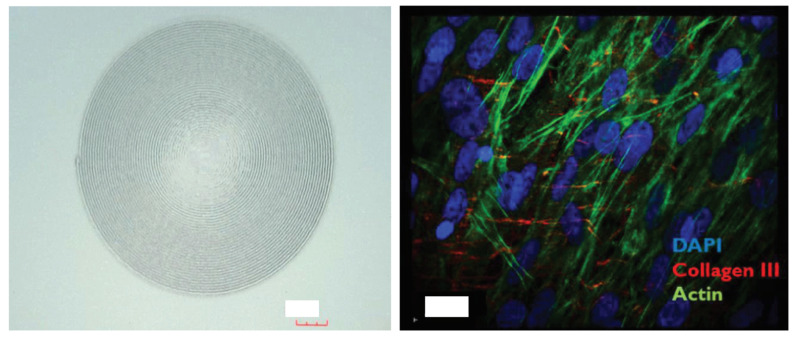
Three-dimensionally printed RHCIII-based corneal stromal scaffolds supporting mesenchymal stem cell proliferation. (**Left**): Optical microscopy image showing a spiral-patterned RHCIII scaffold, scale bar = 600 μm. (**Right**): Immunofluorescence staining revealing MSC adhesion, actin cytoskeletal organization, and collagen type III expression within the printed construct, scale bar = 20 μm. Reproduced from [60] with permission of Elsevier B.V. under CC BY-NC-ND 4.0 license.

In this line, Das et al. (2015) evaluated the supramolecular structures in cell-laden 3D constructs using silk and gelatin as the principal polymer matrix while encapsulating hTMSCs in alginate. After cell incubation, the bioink was enzymatically crosslinked, promoting multilineage differentiation of the encapsulated stem cells and maintaining a stable 3D structure with high cell viability for over one month, demonstrating remarkable cell proliferation and differentiation [86]. Subsequently, in 2017, Gibney et al. used additive biomanufacturing to create an RHC type III multilamellar construct co-cultured with corneal mesenchymal stem cells (CMSCs) and keratocytes [60]. In its construction, they stacked 2, 10, and 29 sheets using a custom-designed additive manufacturing system consisting of a nebulizer, an aerosol filtering mechanism, and a bioprinting head. For mechanical stability, the material was crosslinked by a “click reaction” with 1-ethyl-3-(3-diaminopropyl) carbodiimide (EDC) and N-hydroxy-succinimide (NHS). The results indicated that CMSCs proliferated in all the imprinted constructs, regardless of the number of layers, and proliferated more efficiently in those co-cultured with keratocytes. Likewise, superficial and penetrating growth of CMSC was observed in the constructs. Optical coherence tomography (OCT) determined that the thickness for 29 sheets was, on average, 87 µm. A resulting RHCIII multilayered construct supporting mesenchymal stem cell proliferation is depicted in Figure 8.

In another work published in 2020, Mahdavi et al. proposed the regeneration of the corneal stroma using bioprinting with GelMA bioink mixed with corneal stromal cells [87]. Utilizing visible light-based stereolithography, the authors printed a dome-like structure, testing two GelMA concentrations (7.5 and 12.5 wt%) for bioprinting. They observed that 12.5% GelMA was stiffer than 7.5% GelMA, making it easier to handle and more closely resembling the native corneal stroma in terms of water content and optical transmission. By evaluating the cell proliferation and the gene and protein expression of human corneal stromal cells encapsulated in the bioprinted scaffolds, it was established that cell cytocompatibility in the 12.5% GelMA scaffolds was significantly higher than in the 7.5%, scaffolds, where in both cases, the cells were integrated and grew within the scaffolds. An increase in the gene expression of collagen type I, lumican, and keratan sulfate was also observed in cells grown on GelMA scaffolds at 12.5% compared to the control (cells grown on plastic culture dishes).

The corneal stromal model has also enabled the creation of in vitro study systems; such is the case in the work performed by Kutlehria et al. (2020), who designed high-performance 3D-printed corneal equivalents to address the need for in vitro models [88]. Utilizing a digital 3D cornea model, the biomaterial was fabricated using sodium alginate, gelatin type B, and type I bovine collagen solution-Fibrocol with a CELLINK Bio X bioprinter. To maintain the curvature of the cornea, they developed a support structure through stereolithography, allowing the printing of 6 to 12 corneas at a time. Human corneal keratocytes (HCKs) were incorporated into the bioink, and corneal stromal equivalents were printed. The printed structures were crosslinked with calcium chloride (100 mM), washed with Hank’s balanced salt solution, and incubated at 37 °C in fibroblast medium. The printed corneas maintained their structure, integrity, and transparency. Cell viability and marker expression analysis demonstrated that the HCKs maintained high viability (>95%) after two weeks.

### 3.3. The Endothelium in 3D Bioprinting

**Figure 9:**
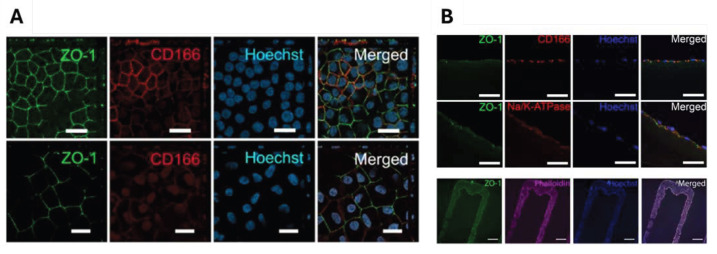
Cell viability of human pluripotent stem cell-derived corneal endothelial. Immunofluorescence images displaying tight junction formation and endothelial marker expression: ((**A**), top) when seeded on the bioink based on hyaluronic acid crosslinked with hydrazone, scale bar = 20 μm; ((**A**), bottom) without bioink, scale bar = 20 μm; and (**B**) controlled pattern bioprinting of hPSC-CEnCs demonstrating high viability post-printing, scale bar = 500 μm. Reproduced from [49] with permission of Springer Nature under CC BY 4.0 license.

The methods used to recreate the corneal endothelium through bioprinting are limited compared to those aimed at creating the epithelium or stroma [89]. Nevertheless, existing methods include developing bioinks, utilizing specific cells, and creating Descemet’s membrane (the basement membrane of the corneal endothelium). In this context, Kim et al. (2018) utilized genetically modified human endothelial cells to overexpress ribonuclease 5, which enhanced their proliferation and survival capacity in a bioink deposited by extrusion onto a decellularized amniotic membrane substrate, demonstrating integration and functional recovery in animal models [90]. Using extrusion-based bioprinting, Grönroos et al. (2024) demonstrated the viability of pluripotent corneal endothelial cells derived from human stem cells with a bioink based on hyaluronic acid crosslinked with hydrazone. In their work, Grönroos found that the structures resembled native endothelial cells and expressed characteristic phenotypic markers such as ZO-1 and Na^+^/K^+^ ATPase, see Figure 9 [49].

### 3.4. Other Approximations in Corneal 3D Bioprinting

**Figure 10:**
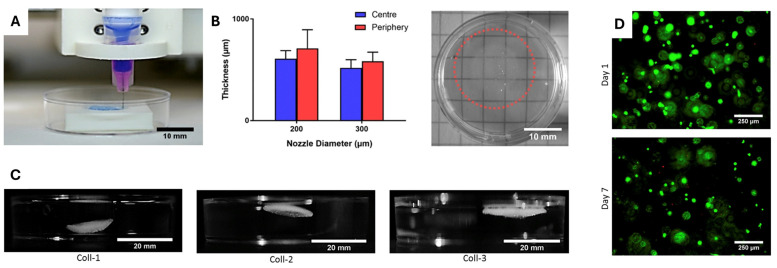
Workflow and results of 3D bioprinting of corneal stromal constructs using support structures and optimized composite bioinks. (**A**) Printing process of corneal structures using FRESH support. (**B**) Transparency evaluation and correlation between nozzle size and construct thickness. (**C**) Final corneal constructs printed with composite collagen–alginate bioinks. (**D**) Live/dead staining demonstrates high keratocyte viability after 7 days. Adapted from [91] with permission of Elsevier B.V. under CC BY 4.0 license.

Despite efforts to generate new implants that mimic a native cornea, most studies have also focused on creating new bioinks and more efficient and effective bioprinting methods. In this regard, Isaacson et al. (2018) used a bioink based on sodium alginate, methacrylate collagen type I, and corneal keratocytes to bioprint a stromal equivalent [91]. The geometry was obtained using the “Finite Element Method” based on elevation data from actual corneal topography, previously described by Simonini and Pandolfi [92], and captured with a Scheimpflug rotating camera and a Placido disk. Bioprinting was performed using two extruders with nozzles of 200 and 300 µm in diameter, and material crosslinking was conducted with calcium chloride. The obtained matrix featured a curved, intersecting structure between sheets, validating the potential generation of a mimetic cornea scaffold from 3D modeling and bioprinting technologies. Cell viability was evaluated for up to 7 days post-impression, with no difference established compared to the control group, and cell adhesion was achieved. Some of the results obtained by the authors, as an approach to full-thickness corneal bioprinting using supporting structures, are shown in Figure 10.

On another note, Kim et al. (2019) demonstrated that applying a controlled cutting force through different nozzle sizes of 3D printers allowed manipulation of the collagen fibers’ organization in the printed structure, resulting in cell alignment and structural organization similar to that of the native human cornea [93]. To this end, the decellularized extracellular matrix of bovine corneal stroma and keratocytes was used as bioink. They then created a lattice-shaped laminar structure analogous to the macrostructure of a mature and transparent cornea. Four weeks after grafting onto New Zealand rabbit corneas (in vivo), the grafts integrated into the surrounding tissue, with no collagen enzymatic degradation observed. Similarly, Duarte et al. (2019) integrated fundamental tissue science parameters using the drop-on-demand (DoD) strategy [94]. They developed a layer-by-layer biomaterial that considers the rheological properties of the native cornea by bioprinting collagen type I and agarose hydrogel. They introduced corneal stromal keratocytes (CSKs) into collagen type I and agarose medium, placing them in a humidified incubator at 37 °C. Evaluation of cell viability showed differentiation from the expected dendritic phenotype of keratocytes seven days after bioprinting, demonstrating cell adhesion and differentiation capacity for potential corneal implant generation.

In 2019, Zhang et al. considered another approximation by employing a mixed extrusion and a digital light-processing printing system they created [95]. They used data from the Pentacam topographer (Oculus, Germany) along with a complex mathematical adjustment for the cornea design. The bioink consisted of methacrylated gelatin, sodium alginate, and human corneal epithelial cells, resulting in a structure resembling a native cornea. The printed corneal substitute was assessed through biomechanical analysis, weight measurements, structural integrity, and fitting. The results indicated that achieving highly transparent, high-water-content curved films with geometric characteristics modeled after the human cornea is possible. Although this work concentrated on geometric control and precision manufacturing, the bioprinting material attained a cell viability greater than 80% and was uniformly dispersed throughout the construct. In a similar vein, Zhong et al. built a dynamic light processing (DLP) bioprinter to study the extracellular matrix-dependent changes in limbal stem cells (LSCs) [96]. The DLP bioprinter, through its spatio-temporal control in the printing process, allowed for the selective encapsulation of LSCs on a micrometer scale. For this, they used rabbit (rbLSCs) and human (hLSCs) limbal stem cells in either gelatin methacrylate (GelMA)- or hyaluronic acid glycidyl methacrylate (HAGM)-based hydrogels, with lithium phenyl-2,4,6-trimethylbenzoylphosphinate (LAP) as the photo-polymerizer. Regarding cell viability, hLSCs showed similar viability after 5 days of incubation in both matrices; however, rbLSCs exhibited greater viability in GelMA than in HAGM. Therefore, confirming that rbLSCs were present in the GelMA matrix and not in HAGM enabled them to leverage the potential of the DLP bioprinter by constructing double extracellular matrix models within the same construct. This ability facilitated spatial differentiation of active and quiescent cell states of the LSCs, which is crucial since these cells alter their proliferative states in response to corneal lesions [97].

Another area of interest is the promotion of cellular regeneration through transfection processes. An example of this is the work published by Kim et al. (2018), in which they developed a culture method for in vivo poorly proliferating human corneal endothelial cells (HCECs) on a lyophilized amniotic membrane using bioprinting [90]. These cells were transfected to overexpress ribonuclease (RNase) 5, a protein associated with angiogenesis and cell survival processes, to enhance their proliferation and survival capacity. A gelatin and endothelial cell-based bioink was extrusion-deposited onto a layer of decellularized bovine amniotic membrane, which acted as Descemet’s membrane. Subsequently, the constructs were implanted into rabbit corneas. In vivo results demonstrated that grafts modified with RNase 5 exhibited higher expression of the Na^+^-K^+^ ATPase pump and a greater number of cells than grafts from the control group. This suggests that HCECs with RNase 5 proliferate better, being almost normal in transparency and superior to the control without RNase 5 after four weeks. No inflammatory fibrinogens were observed in the anterior chamber, and post-surgery corneal edema decreased considerably for grafts with and without RNase 5 compared to an acellular control. Thus, this study established that using genetically modified HCECs with RNase 5 may be a viable option to improve cell proliferation and function of corneal grafts in transplants.

### 3.5. In Vivo Assessment of 3D-Bioprinted Corneas

Concerning the development of 3D-printed scaffolds for corneal tissue engineering, several materials and methods have been employed for in vivo studies. Kim and colleagues (2019) developed a bioink derived from a decellularized corneal stroma extracellular matrix and used extrusion-based 3D printing to control the spatial orientation of collagen fibrils. This approach allows for the precise structuring of the bioink to mimic the natural alignment of collagen in the corneal stroma. In a rabbit model, the results showed that after 4 weeks, the collagen fibrils had remodeled along the printing path due to the controlled architecture and biomimetic properties of the 3D-printed bioink [93].

Other techniques, such as stereolithography, were evaluated by He and colleagues (2022) through the design of a hydrogel-based gelatin methacrylate (GelMA) and long-chain poly(ethylene glycol) diacrylate using LAP as a photoinitiator (with a wavelength of 405 nm). A corneal construct with two layers of cell-laden material was used in a rabbit model of anterior lamellar keratoplasty, promoting corneal and stromal regeneration through biointegration of the implanted scaffold with native tissue after 4 weeks [67]. Further studies by Zhang and colleagues (2023) address the gap between mechanical and biocompatible properties by introducing a decellularized extracellular matrix (CECM) and GelMA as bioink. These results in the rabbit model led to a healing rate of around 93.5% at 28 days post-operation, promoting the restoration of matrix thickness and tissue transparency, in contrast to the corneal defect group, which may lead to corneal scarring. Nevertheless, understanding the surrounding biophysical environment requires further analysis to promote cell differentiation and provide functionalized tissue after cell restoration [98].

The research by Nie and colleagues (2024) highlights the surrounding biophysical environment. Their study introduces an extrusion 3D-printed bilayer scaffold in a gelatin/alginate matrix loaded with recombinant human epidermal growth factor (rhEGF) and Trichostatin A (TSA), a histone deacetylase inhibitor. This scaffold provides an optimal microenvironment for cornea regeneration, maintaining optical transparency and suture resistance, regulating epithelial repair, and minimizing scar formation by playing a critical role in the enhancement of proliferation and migration of rabbit cornea epithelial cells (rCECs) [99].

### 3.6. Traditional Scaffolds vs. Bioprinted Biomaterials for Corneal Tissue

The materials discussed in the previous sections have demonstrated their versatility and potential for use in corneal bioprinting. However, another key consideration is the difference between employing these biomaterials for traditional scaffold construction and using a bioprinter to create functional corneal constructs. In this regard, Balters et al. (2023) compiled a list of biomaterials used in corneal bioprinting bioinks, which aligns with those highlighted in the earlier sections of this review. Their analysis shows that the most frequently employed materials include GelMA (5), hyaluronic acid derivatives (3), collagen (4), gelatin (3), alginate (3), chitosan (1), and decellularized extracellular matrices (2). Notably, these materials require a combination of other components and crosslinkers to strengthen the bioprinted structure [100].

For scaffold construction, GelMA stands out as an excellent option because it closely mimics the corneal stroma, regardless of the source of gelatin B used in its synthesis [101]. Its mechanical properties can be optimized by adjusting the hydrogel concentration and crosslinking conditions, such as light intensity and exposure time [102], as well as the temperature and the flow rate of methacrylic anhydride incorporation [103]. GelMA also offers transparency and high biocompatibility. In this vein, Bektas and Hasirci et al. (2020) reported achieving mechanical strength of approximately 20 kPa in a printed structure, which is close to the 27 kPa reported for native corneas [104]. When applied in scaffolds, even higher values have been reported for compression tests, reaching 2.045 MPa with GelMA prepared from Sigma-Aldrich gelatin B and 1.564 MPa for GelMA commercialized by Halvet, with the possibility of achieving even higher values [101].

Nevertheless, in addition to the advantages described here for the 3D bioprinting technique, several limitations related to tissue engineering must be considered. For instance, a drawback is the limited range of printable biomaterials that can simultaneously support cell viability, structural fidelity, and optical transparency, often requiring careful optimization and the incorporation of additional photocrosslinkers compared to the molding/casting [105] or micropatterning [106] techniques. Consequently, photosensitizers could alter the protein and lipid structures of the additive molecules present in the bioink [107]. Moreover, achieving standardization across bioink formulations remains a significant challenge due to the competing demands of rheological and biological performance. Balancing mechanical strength for shape retention with sufficient softness for cell encapsulation and migration is particularly complex, as overly stiff hydrogels may compress cells and restrict their behavior. Additionally, the resolution is minimal, with a minimum feature size generally exceeding 100 µm, which can affect the biomimetic organization of the construct [108]. These limitations highlight that although 3D bioprinting holds considerable promise, its practical implementation for corneal applications still requires significant optimization.

## 4. Conclusions

Vision is the most critical human sense; however, around 2.2 billion people worldwide are blind due to visual impairments. Corneal blindness is one of the leading causes of this issue. It is one of the most common consequences that can be treated by replacing the damaged and dysfunctional tissue with functional corneal tissue from a donor. However, the availability of suitable donors significantly falls short of meeting the demand for transplantation.

In this context, 3D printing technology has disrupted the biomedical sciences, particularly in tissue engineering, where established methodologies have been adapted for biomedical purposes to generate various types of tissue. In relation to the cornea, multiple attempts have been made to replicate the native human cornea using different bioinks (comprising biopolymers, additives, crosslinkers, and cells), 3D bioprinters, and software/approximations. These approaches have achieved promising results in certain animal models, including transparency, biocompatibility, cell proliferation, adhesion, a non-exacerbation of the inflammatory response, and partial vision recovery.

While various 3D bioprinting techniques have demonstrated promising results in corneal regeneration, significant limitations and challenges remain before these advancements can be clinically translated. Among them, most studies on the cornea topic have been conducted using 3D bioprinting equipment created or modified by the authors themselves, hindering the development of advancements in a reproducible manner on a larger scale. Similarly, the use of different reagents and cells, along with the chosen printing parameters, complicates the establishment of the correct formulation for a corneal acceptor. Furthermore, it is essential to consider that challenges remain in designing organs and tissues related to their rheological and mechanical properties as well as their chemical and biological characteristics, which are necessary to control the corneal implant’s chemical, physical, and biological signaling.

Finally, further studies are necessary to evaluate the potential rejections that implants may experience, using lower-cost bioprinting methods that ensure universal access to these treatments in the future. Nonetheless, corneal 3D bioprinting is still an evolving field with significant progress needed. The efforts made to date have enabled the generation of functional and cytocompatible implants, with some attempts extending to animal models in recent years, demonstrating the application potential of imprinted synthetic corneal constructs.

## Data Availability

No new data were created or analyzed in this study.
